# Holistic nursing competence and clinical learning experience among professionally active nursing students: a cross-sectional study

**DOI:** 10.3389/fmed.2026.1915951

**Published:** 2026-09-25

**Authors:** Magdalena Brodowicz-Król, Paweł Więch, Maria Malm, Katarzyna Wiśniewska, Andrea Sollárová, Grzegorz Józef Nowicki

**Affiliations:** 1 Faculty of Health Sciences and Psychology, Collegium Medicum of the University of Rzeszów, Rzeszów, Poland; 2 Department of Medical Informatics and Statistics with e-Health Lab, Medical University of Lublin, Lublin, Poland; 3 Faculty of Health Sciences, Radom Higher School, Radom, Poland; 4 Department of Nursing, Faculty of Social Sciences and Health Care, Constantine the Philosopher University in Nitra, Nitra, Slovak Republic; 5 Department of Health Care Sciences, Faculty of Humanities, Tomas Bata University in Zlin, Zlin, Czech Republic; 6 Department of Family and Geriatric Nursing, Faculty of Health Sciences, Medical University of Lublin, Lublin, Poland

**Keywords:** clinical competence, education, graduate, holistic nursing, nursing, students

## Abstract

**Background:**

The rapid development of healthcare requires nursing practitioners to continually enhance their skills and adopt a holistic approach. Education is a key component of this process, and is linked with active professional work. The effectiveness of this education, however, is determined by the psychosocial factors of the clinical practice setting. The aim of this study is to assess the relationship between the level of holistic nursing competencies and the perceived quality of clinical practice among nursing students combining education with professional work.

**Methods:**

The cross-sectional study was carried out among a group of 293 practising nurses (mean age 42.9 years, mean length of service 16.5 years) enrolled in a master's degree program. The following two tools were utilised: Holistic Nursing Competence Scale (HNCS) and Ascent to Competence Scale (ACS).

**Results:**

The participants showed a high level of holistic competencies (Me = 5.67), with the “Ethically-oriented practice” and the “Professional development” subscales receiving the highest scores (Me = 6.0). In terms of clinical setting, the “Learning and Competence” subscale obtained the highest score (Me = 4.0), while the “Belongingness” subscale was given the lowest score (Me = 3.71). The sociodemographic variables had no significant impact on the variables under study (*p* > 0.05). Correlation analysis revealed positive and statistically significant relationships between all HNCS and ACS subscales. The total HNC score showed the highest correlation with the ACS “Learning and Competence” subscale (*ρ* = 0.506; *p* < 0.001).

**Conclusions:**

The study demonstrated a positive and statistically significant association between the quality of the clinical practice environment and the level of holistic nursing competence. Among the examined factors, learning opportunities within the clinical setting emerged as the factor most strongly associated with holistic competence.

## 1 Introduction

The professional competence of nurses is one of the key elements influencing healthcare quality, patient safety and healthcare system effectiveness. However, contemporary nursing requires far more than merely following clinical procedures correctly. Nowadays, nurses are expected to make clinical decisions, think critically, engage in interdisciplinary collaboration, follow ethical principles, provide health education, as well as pursue continuous professional development. As a result, the idea of holistic nursing competence (HNC), which includes the integration of knowledge, practical skills, professional attitudes and clinical judgment, is becoming increasingly important (1–4).

The holistic model of nursing competence is based upon a critical review of the literature on nursing competence conducted by Takase and Teraoka (5). The authors observed that the dominant approach to competence in the literature focuses exclusively on the clinical and technical aspects, while ignoring other crucial issues related to professional nursing practice (6). In order to address this gap, they proposed a multidimensional model that incorporates five key domains: general aptitude, understood as the basic personal traits and day-to-day professional behaviours that form the foundation for safe practice; staff education and management, which involve the coordination of educational activities and human resource management; ethically-oriented practice, which encompasses ethical decision-making and respect for patient rights; nursing care in a team, which includes effective collaboration and conflict resolution; professional development related to self-directed learning and career planning (5, 7). According to this model, a truly competent nurse is a person who possesses not only advanced clinical skills but also the ability to lead a team, make ethical decisions, interact in groups and regularly develop professional skills. According to the literature, assessing clinical competence in nursing remains a methodological challenge due to its multifaceted and dynamic nature. So far, no single universal standard for assessing nursing competence has been produced, and many of the available tools focus on technical skills while ignoring ethical, interpersonal and reflective aspects. Therefore, a holistic model for assessing professional competences is recommended (2).

The quality of the clinical learning setting has a strong impact on the development of professional competence in the nursing profession. Clinical practice is a key component of nursing education as it allows the integration of academic knowledge with professional practice and the formation of a professional identity. In connection with the above, Levett-Jones and Lathlean (8) produced a conceptual framework for the Ascent to Competence theory. This concept is founded on research into nursing students’ clinical practice experiences, as well as the psychological theory of belongingness (9). This idea holds that students develop professional competence through a hierarchical process that relies on meeting subsequent needs (10). At the bottom of this pyramid is the need for safety, both physical and psychological, followed by the need for belonging, that is a sense of being accepted and respected as a member of the clinical team. A healthy self-concept as a competent future nurse can only be developed after meeting these fundamental needs. This, in turn, paves the way to successful learning and, ultimately, the achievement of relevant competence. According to research, positive clinical experience increases students’ confidence, helps them acquire professional competence and prepares them to practice independently (11, 12).

A particularly significant aspect of clinical experience is the sense of belongingness within the treatment team. Nursing students who feel accepted and supported in the clinical setting are more motivated, engaged in the learning process and better-prepared for their professional careers. In turn, a lack of support, marginalisation, or a sense of exclusion might have a negative impact on competency development, stress levels and educational satisfaction (13, 14). The quality of the clinical setting is strongly related to nursing student clinical competency. Mentoring support, the ability to actively participate in patient care, constructive feedback, and positive interpersonal relationships all associated with the development of professional competence and sense of professional effectiveness (11).

These two theoretical concepts, that is the holistic model of competence and Ascent to Competence, are closely linked in a way that is essential to understand the nursing education process. Following the combination of these theories in terms of clinical education, it becomes clear that the clinical practice setting establishes the basis for the development of both clinical competencies and more comprehensive aspects of professional practice by satisfying students’ need for belongingness. In other words, a student who feels welcomed and accepted by the team not only learns technical skills more successfully, but also develops a professional ethical attitude, managerial abilities and a dedication to continuing professional development. On the other hand, a lack of sense of belongingness can impede the development of holistic competence, limiting students to merely following procedures without a deeper comprehension of the care-related aspect (15–18).

Despite increased interest in nursing competence, to date, most studies have focused on the effects of professional activity on undergraduate students or newly employed nurses. There is still a limited number of studies examining the relationship between the perceived quality of clinical experiences and the level of holistic professional competence among practising nurses that intend to enhance their competences by pursuing a master's degree. In particular, very little research has been carried out within the framework of the Polish nursing education system. The examination of this correlation has significant practical and educational implications. A better comprehension of the factors impacting the development of holistic nursing abilities can help to enhance educational programs, organise clinical practices and create an environment that promotes nurses’ professional development. The findings of such studies can also be utilised to develop initiatives that improve clinical education quality and boost nurses’ professional competencies in an ever-evolving healthcare system. As a result, the aim of this study is to assess the level of holistic nursing competence and the perceived quality of clinical practice among practising nurses pursuing a master's degree in nursing, as well as to examine the correlation between the level of holistic nursing competence and the perceived quality of clinical experiences. The additional aim was to analyse the impact of selected sociodemographic variables on the study variables.

## 2 Materials and methods

### 2.1 Study design and participants

The cross-sectional study was conducted between January and June 2024. A total of 293 individuals took part in the study. The study group consisted of postgraduate nursing students studying at the Medical University of Lublin, the Academy of Zamość and the Radom Higher School. All of the above universities are located in eastern Poland. The sample was selected using purposive sampling, convenience sampling and snowball sampling.

The inclusion criteria were as follows: 1) being enrolled in a master's degree program in nursing at one of the three universities mentioned above; 2) having practised at least six months as a nurse; and 3) providing voluntary and informed consent to take part in the study. The exclusion criteria were as follows: 1) not being enrolled in a master's degree program in nursing, 2) lack of practising as a nurse or practising for less than 6 months and 3) failure to provide informed consent to participate in the study via the electronic form.

The research material was collected through the computer-assisted web interview (CAWI) method. In the first instance, the questionnaire was posted on the “Google Surveys” website. Subsequently, student body representatives obtained a link to the questionnaire's website and a QR code that led to the electronic version of the questionnaire via messaging apps, such as WhatsApp and Messenger. The representatives were instructed to provide these links to all students in their individual classes. Since each year has three student representatives (one chief student representative and two vice student representatives), the link was distributed to all three representatives in order to verify whether the questionnaire had already been provided. A request to share the link was sent at the beginning of each month during which the study was carried out.

### 2.2 Data collection

A structured survey questionnaire, which consisted of two standardised instruments and questions assessing the sociodemographic status of the respondents, was prepared. All questionnaire items were mandatory in the electronic form, and respondents were required to complete every question before submission; therefore, no missing item-level data were present in the final dataset.

#### 2.2.1 Holistic nursing competence scale (HNCS)

The Polish version of the HNCS questionnaire, which was adapted by Brodowicz and Zarzycka, was utilised in the study (17). The HNCS (5) is a tool that assesses nurses’ professional competences, including not only clinical skills, but also ethical, managerial, educational and professional development aspects. The questionnaire is based upon five key components of holistic nursing competence: General aptitude (7 items), Staff education and management (9 items), Ethically-oriented practice (9 items), Nursing care in a team (7 items) and Professional development (4 items). The HNCS tool consists of 36 items that are rated by the respondent on two different 7-point Likert scales. The responses on the “General Aptitude” subscale vary from 1 (never) to 7 (always), whereas the other four subscales range from 1 (cannot do it at all) to 7 (can do it excellently). The final score of the scale is obtained by summing all points from 36 items and ranges between 36 and 252 points. A higher score indicates higher competence among nurses. Cronbach's alpha coefficient for the study group in question was 0.97 for the entire scale, and ranged from 0.90 to 0.94 for the individual subscales.

#### 2.2.2 Ascent to competence scale (ACS)

The second standardised questionnaire utilised in the study was the ACS scale, as adapted into Polish by Brodowicz and Zarzycka (19). The ACS (10) is an instrument utilised to measure nursing student perceptions of the quality of clinical practice. The questionnaire is based upon three key components of the perceived quality of clinical practices: Being welcomed (6 items), Belongingness (17 items) and Learning and competence (13 items). Higher scores imply a higher perceived quality of the clinical practice experience and better conditions for the development of professional competence. This tool can be utilised not only to assess nursing students, but also, as the author suggests, to assess the professional competence of practising nurses. Cronbach's alpha coefficient for the group under study ranged from 0.82 to 0.95 for each subscale.

#### 2.2.3 Socio-demographic variables

The authors created a questionnaire with four closed-ended questions about gender (male/female), age, length of service and place of residence (urban/rural area).

### 2.3 Ethics approval

The study was conducted in accordance with the Declaration of Helsinki. Ethical approval was obtained from the Bioethics Committee at the Medical University of Lublin (decision number: 148/2023). The study involved human subjects, and all participants provided electronic informed consent prior to their participation. All procedures were carried out in compliance with relevant institutional and national regulations.

### 2.4 Statistical analysis

Categorical variables were reported as absolute numbers and percentages. Continuous variables were presented as median (interquartile range, IQR). The Shapiro–Wilk test was utilised to assess conformity with a normal distribution. The distribution of responses between the HNCS and ACS scores and the analysed variables was compared through the Mann–Whitney test. The Spearman correlation coefficient was applied to assess the relationship between the HNCS and ACS score. Statistical analyses were performed using IBM SPSS Statistics for Windows, Version 28.0 (Armonk, NY: IBM Corp) and R Core Team (2024) (R: A Language and Environment for Statistical Computing; R Foundation for Statistical Computing, Vienna, Austria). For all statistical tests, a p-value < 0.05 was considered statistically significant. For the correlation analyses, a Bonferroni correction for multiple comparisons was applied (18 comparisons; adjusted significance level: *p* < 0.0028). A *post hoc* power analysis was performed using G*Power 3.1.9.7 (Heinrich Heine University Düsseldorf, Düsseldorf, Germany) based on the smallest observed correlation coefficient, the total sample size, and the Bonferroni-adjusted significance level.

## 3 Results

### 3.1 Characteristics of participants

The study included 293 practising nurses enrolled in a master's degree program in nursing. The mean age was 42.93 (± 9.93) years. The youngest respondent was 26 years old and the oldest respondent was 61 years old. The average length of experience in the nursing profession was 16.56 (±12.33) years. The shortest length of professional practice as a nurse was 8 months, while the longest was 40 years. The majority of the study group consisted of women (96.59%, *n* = 283) and urban residents (81.57%, *n* = 239).

### 3.2 Assessment of the level of holistic nursing competence in the study group - results of the assessment of the HNCS scale

Table 1 shows the results of the assessment of the level of holistic nursing competence in the study group in question. The median total HNC score was 5.67 (Q1–Q3: 5.67-6.11). The “Ethically-oriented practice” and “Professional development” subscales obtained the highest median score, while the “Staff education and management” and “General aptitude” subscales received the lowest median scores. None of the sociodemographic variables under study (age, length of service, sex and place of residence) had a significant impact on the level of holistic nursing competence (Supplementary Table S1).

**Table 1:** Level of holistic nursing competence in the study group.

HNCS	Me (Q1-Q3)
Subscale 1: General aptitude	5.43 (4.78-6.0)
Subscale 2: Staff education and management	5.44 (4.78-6.0)
Subscale 3: Ethically-oriented practice	6.0 (5.44-6.56)
Subscale 4: Nursing care in a team	5.86 (5.14-6.29)
Subscale 5: Professional development	6.0 (5.25-6.50)
Total HNC	5.67 (5.08-6.11)

HNC, Holistic Nursing Competence Scale; Me, median; Q1, first quartile; Q3, third quartile.

### 3.3 Assessment of nursing student perceptions of clinical practice quality using the ACS instrument

Table 2 shows the results of the ACS scale assessment in the study group of students. The assessment of the perceived quality of clinical practice through the ACS scale showed that nursing students rated the “Learning and competence” subscale the highest (Me = 4.00; Q1–Q3: 3.62–4.31), then “Being welcomed” (Me = 3.83; Q1–Q3: 3.33–4.33), with “Belongingness” being the lowest (Me = 3.71; Q1–Q3: 3.18-4.18). The sense of “Being welcomed” and “Belongingness” received the most different responses, which may indicate varied student experiences regarding integration into the clinical team. None of the sociodemographic variables under study (age, length of service, sex and place of residence) had a significant impact on the assessment of the perception of the quality of clinical practice (Supplementary Table S2).

**Table 2:** Descriptive statistics of the ACS among nursing students.

ACS	Me (Q1-Q3)
Subscale 1: Being welcomed	3.83 (3.33-4.33)
Subscale 2: Belongingness	3.71 (3.18-4.18)
Subscale 3: Learning and competence	4.0 (3.62-4.31)

ACS, Ascent to Nursing Competence Scale; Me, median; Q1, first quartile; Q3, third quartile.

### 3.4 Correlations between holistic nursing competence and clinical practice quality perceptions

Table 3 shows the correlation between holistic nursing competence and the assessment of perceived clinical practice. According to correlation analysis, all HNCS and ACS subscales had positive and statistically significant relationships. The “Learning and competence” subscale (*ρ* = 0.506; *p* < 0.001) showed the greatest correlation with the total HNC score, followed by the “Belongingness” subscale (*ρ* = 0.359) and the “Being welcomed” subscale (*ρ* = 0.314). The weakest correlation was observed between the “Professional Development” and “Being Welcomed” subscales (*ρ* = 0.201; *p* = 0.001). In summary, as compared to the other two ACS subscales, the “Learning and competence” item of the ACS scale consistently demonstrated stronger correlation with holistic nursing competence. This implies that perceived learning opportunities during clinical practice may be particularly important for the development of holistic nursing competence. A *post hoc* power analysis based on the smallest observed correlation coefficient (*ρ* = 0.201), a sample size of 293, and the Bonferroni-adjusted significance level (*α* = 0.0028) indicated an achieved statistical power of 0.68.

**Table 3:** Correlation between holistic nursing competence and the assessment of clinical practice.

HCNS	ACS								
	Being welcomed			Belongingness			Learning and competence		
	*ρ*	95% CI	*p*	*ρ*	95% CI	*p*	*ρ*	95% CI	*p*
Staff education and management	0.253	0.134-0.369	<0.001	0.341	0.228-0.445	<0.001	0.455	0.350-0.556	<0.001
Ethically-oriented practice	0.318	0.206-0.430	<0.001	0.298	0.182-0.405	<0.001	0.422	0.310-0.527	<0.001
General aptitude	0.305	0.190-0.411	<0.001	0.345	0.234-0.452	<0.001	0.424	0.317-0.520	<0.001
Nursing care in a team	0.295	0.180-0.407	<0.001	0.312	0.197-0.414	<0.001	0.453	0.347-0.551	<0.001
Professional development	0.201	0.079-0.314	0.001	0.242	0.124-0.353	<0.001	0.414	0.302-0.514	<0.001
Total HNC	0.314	0.204-0.424	<0.001	0.359	0.245-0.462	<0.001	0.506	0.402-0.596	<0.001

HNC, Holistic Nursing Competence Scale; ACS, Ascent to Nursing Competence Scale; The significance level was adjusted using the Bonferroni correction: *α* = 0.05/18 = 0.00278.

## 4 Discussion

This study contributes to the existing body of knowledge about the development of nurses’ professional competence by demonstrating that these competencies should be viewed as a multidimensional phenomenon that is strongly dependent on the environmental context, rather than as a collection of technical and procedural skills. The employment of two complimentary instruments, the Holistic Competence Scale and the Ascent to Competence Scale, allowed for a multidimensional analysis of respondents’ internal competence and the environmental conditions that may promote their further growth. Our study findings not only confirm the theoretical assumptions of the models proposed by Takase and Teraoka (5) and Levett-Jones and Lathlean (8, 10), but they also add new value by examining these correlations within the specific study group, which combines the role of a student with active professional work as a nurse.

The HNCS scale results show that the nurses under study assessed their overall holistic competence as relatively high. The “Ethically-oriented practice” and “Professional development” subscales obtained particularly high scores, indicating that respondents were personally committed to ethical aspects of care as well as awareness of the need for continuous professional development. However, it should be noted that the particularly high scores observed in the “Ethically-oriented practice” subscale may have been partly influenced by social desirability bias. Given the nature of the questions, respondents may have been inclined to overestimate their ethical competencies in order to align with professional norms and expectations, which could have inflated the reported levels in this dimension. These results are consistent with the findings reported by Jafari Rahim Abad et al. (20) who also discovered moderately high levels of HNC in a group of 784 nurses who took care of elderly persons, with ethical competence emerging as the factor most strongly associated with holistic competence. Additionally, this view of competence is consistent with Takase and Teraoka's (5) definition of holistic nursing competence, which covers a nurse's ability to effectively use a set of attributes, such as personal traits, professional attitudes, values, knowledge and skills, in order to fulfil professional responsibilities through practice. On the other hand, the HNC scores obtained in our study were greater than those reported in prior research involving actively practising nurses (21) or nursing students (22). The higher scores obtained in the group under study can be attributed to its unique characteristics - as the participants were not undergraduate students, but experienced professional nurses who consciously chose to pursue a master's degree. As previously reported, length of clinical experience correlates considerably and positively with total HNCS score (5). According to the authors’ research, professional experience is associated with more mature professional attitudes, specifically the ability to make ethical decisions and the desire for self-improvement.

Interestingly, our study results for the “Staff education and management” subscale, which, along with “General aptitude,” obtained the lowest score, are especially notable. The above is supported by the literature: managerial abilities are frequently developed later in the professional career and are not equally available to all nurses, particularly those who do not hold managerial responsibilities. In a study of Turkish nurses, this subscale rated the lowest as compared to other HNCS dimensions (21). The above shows that, in clinical practice, team management and staff education competences are the most requiring targeted assistance and development.

Unlike some earlier research that identified significant relationships between abilities and length of service or age (20), this study revealed no significant correlations with any of the sociodemographic characteristics under study. A possible reason for this discrepancy is the group's very homogeneous characteristics: all respondents are practising professionals pursuing a master's degree. Perhaps the key predictors of holistic competences are not sociodemographic characteristics, but the quality of educational and professional experiences, as well as individual engagement to personal development. Similarly, gender had no effect on HNCS results, which could be attributed to the great majority of women in the study sample (96.59%). It is worth mentioning, however, that a validation study of the Persian version of the HNCS discovered no gender-related measurement bias, implying that the instrument itself is not gender biased (23).

The assessment of the quality of clinical practice through the ACS scale showed that students rated the “Learning and competence” subscale the highest, followed by “Being welcomed”, with “Belongingness” being the lowest. This result implies that while respondents recognise the high educational value of the clinical setting, they do not necessarily feel full social and professional integration within therapeutic teams involved in their training. Our findings are consistent with those found in Polish validation studies of the ACS scale (19) and in other papers utilising this tool (24). The sense of “Belongingness” and “Being welcomed” showed the greatest variation in responses (as determined by the interquartile range), which may indicate that student experiences with integration into the clinical team are highly variable and dependent on the organisational context. The “Belongingness” subscale, which measures the sense of being an accepted and valued member of the team, obtained the lowest score, which is especially concerning. The Ascent to Competency thesis holds that a student's need for belongingness must be addressed before a person can completely focus on learning and acquiring competency (8). Meanwhile, a lack of sense of belongingness may have a detrimental impact not just on learning effectiveness, but also on motivation, self-confidence, as well as readiness to take on occupational challenges.

In our study group, which combines the responsibilities of student and employee, this “dual” identity may even complicate the integration process: staff may regard students as “temporary” guests or even as persons who cannot be fully integrated into the clinical team. However, as our study did not include a comparison group, this interpretation remains speculative and should be treated as a hypothesis for future research rather than a concluded finding. Further studies with comparative designs are needed to examine whether the dual student-employee identity indeed influences belongingness and, if so, under what conditions.

Similarly to the HNCS, none of the sociodemographic variables had a significant influence on the ACS results. The lack of a relationship between age and length of service may indicate that among nurses with many years of professional experience (average length of service: 16.6 years), perceptions of the quality of clinical practice are largely independent of these factors. For these nurses, clinical practice is more of a part of professional development than a basic stage of professional induction.

One of the most significant findings is the presence of positive and statistically significant correlations between all dimensions of holistic nursing competence and the assessment of quality of clinical experience. These results support the assumptions of the Ascent to Competence theory, which states that the development of professional competence is a process that is dependent on the quality of the learning environment and the extent to which the need for belongingness is met. According to Levett-Jones and Lathlean, effective learning in a clinical setting requires a sense of safety and acceptance. Our findings showed a particularly strong relationship between the total HNC score and the “Learning and competence” aspect. The above suggests that a higher assessment of learning opportunities during clinical practices is associated with a higher level of holistic nursing competence. The learning and competency dimension on the ACS scale, as defined by McCoy et al. (10) as the extent to which clinical practices allow students to develop knowledge and skills and achieve competence, appears to be especially important for maintaining and improving holistic competencies among practising nurses. The above is in line with the workplace learning theory, which Takase and Teraoka (5) applied to nursing competence, showing that higher levels of self-assessed competence are correlated with learning via reflection, practice and feedback.

It is worth noting that the “Learning and competence” dimension regularly demonstrated stronger relationships with holistic competence than the other two ACS subscales. This implies that perceived active learning opportunities, constructive feedback and professional challenges are critical for the development of advanced holistic competence. Thus, our study results offer empirical support to the Levett–Jones and Lathlean model, which holds that fulfilment of the need for learning and competence is at the highest level of the development pyramid, and can only be attained upon meeting lower-level needs (safety, belonging and a healthy self-concept).

It is especially important to note the strong correlation between the “Staff education and management” subscale and the ACS dimensions, particularly the “Learning and competence” subscale. This implies that skills connected with personnel management and training improve in workplaces with a strong learning and support culture. The above is in line with Bandura's theory of social learning, which holds that students’ future conduct as teachers and leaders is influenced by how their mentors and supervisors treat them (25). In other words, this study result may indicate that a learning-friendly clinical setting is associated with not only strictly clinical competence, but also competence related to job organisation, team management, and educational activities. Contemporary nursing competency models stress that nurses are increasingly taking on coordinating and leading roles, and that managing care processes is a critical component of professional competence. Of note, Erickson et al. (26) demonstrated that leadership and educational competence are among the most crucial components of modern nursing practice.

In our study, the poorest (but still significant) relationship was observed between “Professional development” and “Being welcomed.” This may imply that the subjective feeling of being welcomed in the clinical team is less significant for the participating nurses’ independent professional development activities, such as extending their qualifications or engaging in professional development projects.

The study results have major practical implications for the organisation of undergraduate and graduate education. Firstly, the authors’ research confirms the importance of creating a positive and supportive learning environment in clinical practice setting, even for experienced nurses who are continuing their education. Clinical practices should be organised in such a way that they maximize possibilities for active learning, reflection and constructive feedback. Secondly, the emphasis on ethical practice and professional development in competence self-assessment suggests that master's degree programs for nurses should prioritise enhancing these dimensions by combining them with real-world clinical experience. Thirdly, the fact that age and length of service had no effect on holistic competence suggests that individual traits (for instance, motivation to develop, self-efficacy) and the quality of the work and learning environment are more important than simply the length of professional experience. As a result, interventions aimed at enhancing holistic competence should be targeted at all nurses, regardless of their level of experience.

The strengths and weaknesses of this study should also be considered. Firstly, two standardised and validated research instruments, which demonstrated high reliability in the study group, were utilised in this study. The inclusion of validated questionnaires improved the reliability of the data and allowed for comparisons with previous studies. Secondly, the study was carried out at three academic centres, which increased the generalisability of the findings. Thirdly, the study group's unique nature, that is practising nurses who made conscious decisions to pursue a master's degree, should be noted and provides substantial insights, as this group is rarely documented in the literature. The majority of research to date have focused on undergraduate students or newly employed nurses.

Despite these strengths, our study has certain limitations. Firstly, the study was cross-sectional, making it impossible to establish cause-and-effect conclusions. Secondly, the sample was selected using purposive sampling, convenience sampling and snowball sampling. Such non-random selection procedures increase the risk of systematic error since they do not ensure representativeness of the full population of practising nursing students. Furthermore, recruitment was carried out through student representatives who distributed invitations via social media platforms (WhatsApp/Messenger). As a result, no denominator — i.e., the total number of eligible students contacted — was available, which prevented the calculation of a response rate and the assessment of non-response bias. This further limits the generalizability of the findings. An *a priori* sample size calculation was not performed because the study employed a non-probability sampling strategy, which also means that the sample size was not statistically justified before data collection. Thirdly, a self-report measure, which carries a risk of error, was employed in the study. Respondents may provide answers in accordance with social expectations (social desirability bias), particularly when asked about ethical competence and professional views. In addition, a subjective self-assessment of competence may not align with an objective clinical assessment made by supervisors or mentors. Moreover, since both instruments were self-report measures administered via the same CAWI method at a single time-point, the study is susceptible to common-method bias, which may have inflated the observed relationships between the variables. It should be acknowledged that confirmatory factor analysis (CFA) was not performed in our sample. We assumed structural invariance of the instruments based on the original validation studies (5, 10) and the Polish adaptation studies (17, 19), in which the factorial structures were confirmed. However, the lack of CFA in the current sample remains a limitation that should be addressed in future research. However, it should be added that self-assessment of competencies is an important psychological construct that determines self-efficacy and motivation to study. Fourthly, the homogeneity of the sample in terms of gender was the subsequent limitation of the study. Although it reflects the real gender structure in the nursing profession in Poland (where men account for around 1-2%), it precludes a study of gender disparities and limits the possibility of transferring the findings to the population of men practising as nurses. Fifthly, the study was conducted exclusively in eastern Poland, hence, both the model of holistic competence and the perception of the quality of the clinical learning environment may be subject to cross-cultural modifications resulting from differences in educational systems, healthcare organisation and social norms. Finally, sixthly, there are potential errors connected with the CAWI data collection method. Although this method ensures anonymity and may reduce the pressure related to social expectations, the lack of direct contact with the interviewer makes it impossible to clarify any doubts concerning the interpretation of the questions. In summary, despite the limitations stated, which are typical for cross-sectional research utilising convenience sampling and self-reports, this study provides valuable information on the association between the clinical learning environment and holistic abilities among practising nursing students. These results should be treated as a contribution to further, in-depth analyses, including longitudinal and intervention studies, which would allow for the drawing of cause–and-effect conclusions.

## 5 Conclusions

The study revealed a positive and statistically significant association between the quality of the clinical practice setting and the level of holistic nursing competence, with perceived learning opportunities in the clinical environment emerging as the factor most strongly associated with competence development. The practising nurses in this study (who are pursuing master's degrees) demonstrate a high level of holistic competence, especially in the areas of ethical practice and professional development, while their skills in personnel management and education require the most support. At the same time, among the measured dimensions of clinical experiences, the sense of belongingness to the clinical team obtained the lowest score, indicating that integration in this area has to be improved. Sociodemographic variables such as age, length of service, gender and place of residence were found to have no significant effect on the level of holistic competence or the assessment of clinical practice quality in the study group. The study results suggest that the development of advanced, holistic competence among practising master's students is significantly associated with the quality of the educational culture of the healthcare institutions where the students complete their clinical practice.

## Data Availability

The raw data supporting the conclusions of this article will be made available by the authors, without undue reservation.
