# Supplementary material for: Holistic nursing competence and clinical learning experience among professionally active nursing students: a cross-sectional study
Source: Front Med (Lausanne). 2026 Sep 25;13:1915951. doi: 10.3389/fmed.2026.1915951 (PMC13649428; doi:10.3389/fmed.2026.1915951)
Supplement: Supplementary file 1 [file Table1.docx]

Supplementary Material

# Holistic Nursing Competence and Clinical Learning Experience Among Professionally Active Nursing Students: A Cross-Sectional Study

## Table S2. Association between selected sociodemographic variables and the assessment of the perceived clinical practice.

| **Variables** | | **Being welcomed** | **Belongingness** | **Learning and competence** |
| --- | --- | --- | --- | --- |
| Spearman's rank correlation | | | | |
| Age | ρ | 0.072 | 0.033 | 0.054 |
|  | 95% CI | [-0.048; 0.195] | [-0.084; 0.152] | [-0.062; 0.168] |
|  | *p* | 0.217 | 0.571 | 0.358 |
| Years of experience | ρ | 0.052 | 0.007 | 0.013 |
|  | 95% CI | [-0.074; 0.176] | [-0.120; 0.130] | [-0.108; 0.133] |
|  | *p* | 0.396 | 0.910 | 0.825 |
| Mann-Whitney U test | | | | |
| Gender: |  |  |  |  |
| Female | Me (Q1-Q3) | 3.83  (3.33-4.33) | 3.71  (3.18-4.18) | 4.0  (3.62-4.31) |
| Male | Me (Q1-Q3) | 3.67  (3.0-4.17) | 3.38  (2.47-3.76) | 3.69  (3.15-4.0) |
|  | Z | 1.122 | 1.318 | 1.295 |
|  | *p* | 0.262 | 0.188 | 0.195 |
| Place of residence: |  |  |  |  |
| Urban | Me  (Q1-Q3) | 3.83  (3.33-4.33) | 3.71  (3.12-4.18) | 4.0  (3.62-4.31) |
| Rular | Me  (Q1-Q3) | 3.83  (3.67-4.17) | 3.68  (3.35-4.24) | 4.0  (3.69-4.38) |
|  | Z | -1.032 | -0.720 | -1.044 |
|  | *p* | 0.302 | 0.471 | 0.297 |

# Note: Data for the Mann–Whitney U test are presented as median (Q1–Q3). Spearman's rank correlation coefficients (ρ) are presented with 95% confidence intervals (CI).
