# Supplementary material for: Holistic nursing competence and clinical learning experience among professionally active nursing students: a cross-sectional study
Source: Front Med (Lausanne). 2026 Sep 25;13:1915951. doi: 10.3389/fmed.2026.1915951 (PMC13649428; doi:10.3389/fmed.2026.1915951)
Supplement: Supplementary file 2 [file Table2.docx]

Supplementary Material

# Holistic Nursing Competence and Clinical Learning Experience Among Professionally Active Nursing Students: A Cross-Sectional Study

## Table S1. Association between selected sociodemographic variables and the level of holistic nursing competence.

| **Variables** | | **Staff education and management** | **Ethically-oriented practice** | **General aptitude** | **Nursing care in a team** | **Professional development** | **Total HNC** |
| --- | --- | --- | --- | --- | --- | --- | --- |
| **Spearman's rank correlation** | | | | | | | |
| Age | ρ | 0.067 | 0.092 | 0.065 | 0.074 | -0.014 | 0.065 |
|  | 95% CI | [-0.053; 0.184] | [-0.028; 0.206] | [-0.053; 0.176] | [-0.040; 0.188] | [-0.129; 0.106] | [-0.052; 0.177] |
|  | *p* |  |  |  |  |  |  |
| Years of experience | ρ | -0.013 | 0.047 | 0.037 | 0.053 | -0.047 | 0.017 |
|  | 95% CI | [-0.138; 0.111] | [-0.075; 0.164] | [-0.088; 0.153] | [-0.070; 0.171] | [-0.171; 0.077] | [-0.108; 0.137] |
|  | *p* |  |  |  |  |  |  |
| **Mann-Whitney U test** | | | | | | | |
| Gender: |  |  |  |  |  |  |  |
| Female | Me (Q1-Q3) | 5.44  (4.78-6.0) | 6.0  (5.44-6.56) | 5.43  (5.57-6.0) | 5.86  (5.14-6.29) | 6.0  (5.25-6.50) | 5.67  (5.08-6.11) |
| Male | Me (Q1-Q3) | 5.28  (4.67-5.44) | 5.78  (4.89-6.11) | 5.21  (4.57-6.0) | 5.43  (5.14-6.57) | 6.50  (5.50-7.0) | 5.58  (4.92-5.94) |
|  | Z | 0.832 | 0.921 | 0.184 | 0.442 | -1.206 | 0.374 |
|  | *p* | 0.406 | 0.357 | 0.854 | 0.658 | 0.228 | 0.708 |
| Place of residence: |  |  |  |  |  |  |  |
| Urban | Me  (Q1-Q3) | 5.44  (4.67-6.0) | 6.0  (5.44-6.56) | 5.43  (4.57-6.0) | 5.89  (5.14-6.29) | 6.0  (5.25-6.50) | 5.64  (5.11-6.11) |
| Rular | Me  (Q1-Q3) | 5.44  (4.89-5.78) | 6.0  (5.33-6.44) | 5.43  (5.0-5.86) | 5.93  (5.0-6.57) | 6.0  (5.0-6.75) | 5.78  (5.08-6.11) |
|  | Z | 0.005 | -0.298 | -0.136 | -0.441 | -0.212 | -0.308 |
|  | *p* | 0.99 | 0.766 | 0.892 | 0.659 | 0.832 | 0.758 |

# Note: No statistically significant associations or group differences were observed (*p* > 0.05). Data for the Mann–Whitney U test are presented as median (Q1–Q3). Spearman's rank correlation coefficients (ρ) are presented with 95% confidence intervals (CI).
